# Impact of dementia caregiving on the labor force participation and lifetime earning of middle age adults

**DOI:** 10.1002/alz70860_101356

**Published:** 2025-12-23

**Authors:** Manying () Cui, Johanna Thunell, Bryan Tysinger, Hanke Heun‐Johnson, Duncan Ermini Leaf, Geoffrey Joyce, Mireille Jacobson, Sidra Haye, Julie M Zissimopoulos

**Affiliations:** ^1^ University of Southern California, Los Angeles, CA, USA

## Abstract

**Background:**

Many persons living with dementia receive care from adult children. Providing care is mentally and physically demanding, and time intensive and may reduce caregivers’ lifetime earnings and wealth. Population estimates of the financial impact of dementia caregiving has been limited by lack of nationally representative, longitudinal data on changes over time of middle‐age adults’ work and time spent caregiving and their parents’ health, and of estimates using rigorous panel data methods.

**Method:**

We used nationally‐representative data from 2012‐2018 waves of the Health and Retirement Study on the demographic, economic and health characteristics of respondents ages 50 to 64 and of their parents/parents‐in‐law (hereafter “parents”). Primary outcomes were caregiving for a parent with dementia and labor force status. We first used fixed effect models to estimate the probability of providing care to a parent with dementia and quantify the association of dementia caregiving and labor supply independent of unobserved differences. We then employed the United States Cost of Dementia Model (USCDM), a validated dynamic microsimulation model, to quantify the impact of dementia caregiving on lifetime earnings and wealth accumulation for a cohort of middle age adults.

**Result:**

We used data from 7,522 respondents and their 12,010 living parents. Fifty four percent of respondents were female and mean age was 58. Sixty seven percent of respondents’ parents were female and mean age was 82.7. About one‐fifth (20.4%) of respondents had a least one parent with dementia. Among respondents who had a parent with dementia, 19.4% were providing care for an average 17.9 hours per week. Among dementia caregivers, 57.5% were in the labor force. A 10% increase in wage rate was associated with a 0.08% decline in the probability of being a dementia caregiver. The likelihood of working was reduced by 3.4% for dementia caregivers relative to non‐caregivers.

**Conclusion:**

Dementia caregiving is common among middle age adult children and reduces their labor supply. For most workers, earnings levels peak at middle age and labor market exits are permanent. Ongoing work employs dynamic microsimulation to quantify the impact of dementia caregiving on lifetime earnings and household wealth accumulation and differences by sex, race, and education.

